# Bilateral Vascular Variations at the Renal Hilum: A Case Report

**DOI:** 10.1155/2012/968506

**Published:** 2012-12-30

**Authors:** Naveen Kumar, Ashwini P. Aithal, Anitha Guru, Satheesha B. Nayak

**Affiliations:** Department of Anatomy, Melaka Manipal Medical College (Manipal Campus), Manipal University, Madhav Nagar, Karnataka, Manipal 576104, India

## Abstract

Imaging technology with its advancement in the field of urology is the boon for the patients who require minimally invasive approaches for various kidney disorders. These approaches require a precise knowledge of the normal and variant anatomy of vascular structures at the hilum of the kidney in terms of their pattern of arrangement and division. The present paper describes a bilateral anomalous arrangement of the structures at the renal hilum as well as their peculiar branching pattern which is of clinical and surgical relevance. Multiple branching of the renal vessels was observed in both kidneys due to which the hila were congested. The right renal artery immediately after its origin divided into 2 branches. The upper branch represented an aberrant artery whereas the lower branch gave 5 divisions. The left renal artery also divided into 2 branches much before the hilum as anterior and posterior divisions. The anterior branch took an arched course and gave 6 branches. The posterior branch gave 3 terminal branches before entering the renal substance. In addition to anomalous hilar structures, normal architecture of both kidneys was altered and the hilum of the left kidney was found on its anterior surface.

## 1. Introduction

Kidneys are a pair of excretory organs situated one on each side of the vertebral column retroperitoneally. Being with a bean shape, it presents thick and rounded superior pole and thin and pointed inferior pole. Renal hilum is deep vertical slit situated in its medial border which lies about 5 cm from the midline opposite the lower border of L1 vertebra. It communicates with the renal sinus within the kidney [1]. According to conventional description in standard anatomy textbooks, at the hilum, usually the renal vein is the anterior mostly with the renal artery posterior to it and the pelvis of kidney lying further posteriorly [2] Various case reports have been published which report possible variations in the arrangement of structures at the hilum. However bilateral anomalous variations of the renal vessels along with abnormal shape of the hilum have not yet been reported.

Knowing the anatomy of the ureteropelvic junction of the kidney is essential for understanding urinary tract disorders and various nephron sparing surgical procedures. The present study describes the bilateral anomalous arrangement of the structures at the hilum of kidney which is of clinical and surgical relevance.

## 2. Case Report

**Figure 1:**
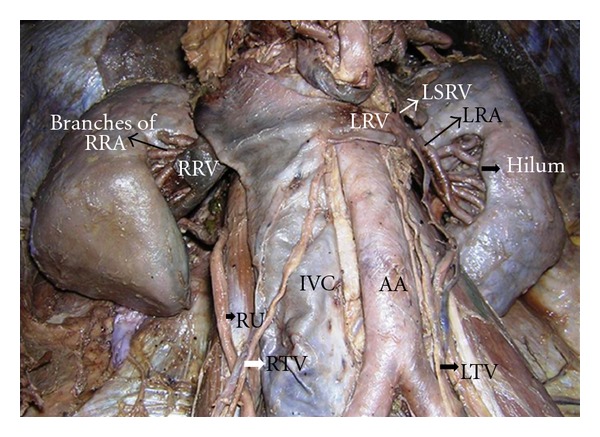
Anomalous vascular branching pattern and their course in the hila of bilateral kidney. IVC: inferior vena cava, AA: abdominal aorta, RU: right ureter, RTV: right testicular vessels, LTV: left testicular vessels, RRV: right renal vein, LRV: left renal vein, and LSRV: left supra renal vein.

During dissection of about 60-year-old male cadaver, we observed anomalous positions and branching pattern of the renal vessels causing a congested renal hilum. The variation was bilateral (Figure 1). The hilar region was dissected carefully and the structures and their relations were clearly defined. The normal architecture of bean shaped kidney was totally distorted bilaterally. Formation of renal pelvis was normal on both kidneys but the variation is as reported below.

### 2.1. On the Right Side

**Figure 2:**
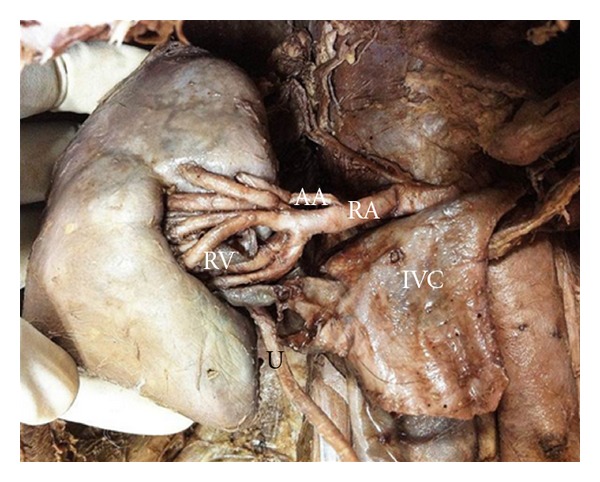
Right kidney: showing congested hilum due to multiple branching pattern of renal artery (RA). AA: aberrant artery, RV: renal vein, IVC: inferior vena cava, and U: ureter.

Renal artery (RA) with its normal origin and course from abdominal aorta divided immediately into 2 branches (Figure 2). The superior branch pierced the upper pole of the kidney without passing through the hilum. It represented the aberrant artery (AA). It gave a thin branch which descended down to the hilum. The inferior branch coursed forward to the hilum and just before entering the substance of the kidney it gave six divisions. The upper 4 branches reached the hilum passing anterior to the renal vein, whereas the lower 2 branches passed posterior to it. The 2 tributaries of renal vein (RV) after emerging from the hilum united to form a single trunk as right renal vein outside the hilum and it drained into the inferior vena cava.

### 2.2. On the Left Side

**Figure 3:**
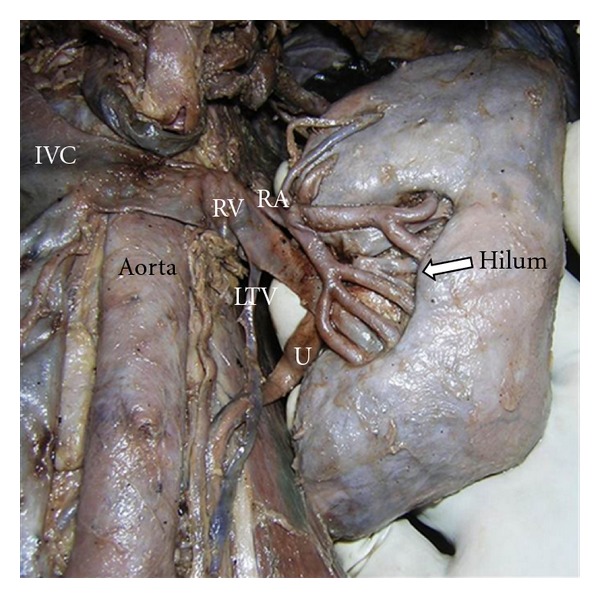
Left kidney: showing congested hilum and distorted shape of the kidney. RA: renal artery, RV: renal vein, IVC: inferior vena cava, U: ureter, and LTV: left testicular vein.

The hilum was wide and situated on the anterior surface instead of its normal anatomical situation in the medial border (Figure 3).

Left renal artery arose from abdominal aorta, before entering the hilum branched into 2 divisions. Anterior division presented an arched course superficial to the tributaries of renal veins and gave 6 branches. The upper 2 branches of it represented the aberrant arteries and entered the upper pole of the kidney. One of the aberrant arteries before piercing the substance of the kidney gave the right inferior suprarenal artery. The posterior division ran behind the renal pelvis and posterior division of renal vein and gave 3 branches. So altogether, 8 branches pierced the renal hilum and 2 branches pierced the upper pole of the kidney.

Anterior and posterior tributaries of renal vein after emerging separately from hilum of the left kidney united to form a single trunk that drained into inferior vena cava. Before the union, the posterior division joined the anterior division in a twisted manner. Anterior division received left testicular vein (LTV). The left suprarenal vein (LSRV) drained into the trunk of the left renal vein. So the arrangement of the structures in the hilum of left kidney from anterior to posterior aspect was anterior division of the renal vein-anterior division of renal artery-renal pelvis-posterior division of renal vein-posterior division of renal artery (A-V-P-V-A).

**Figure 4:**
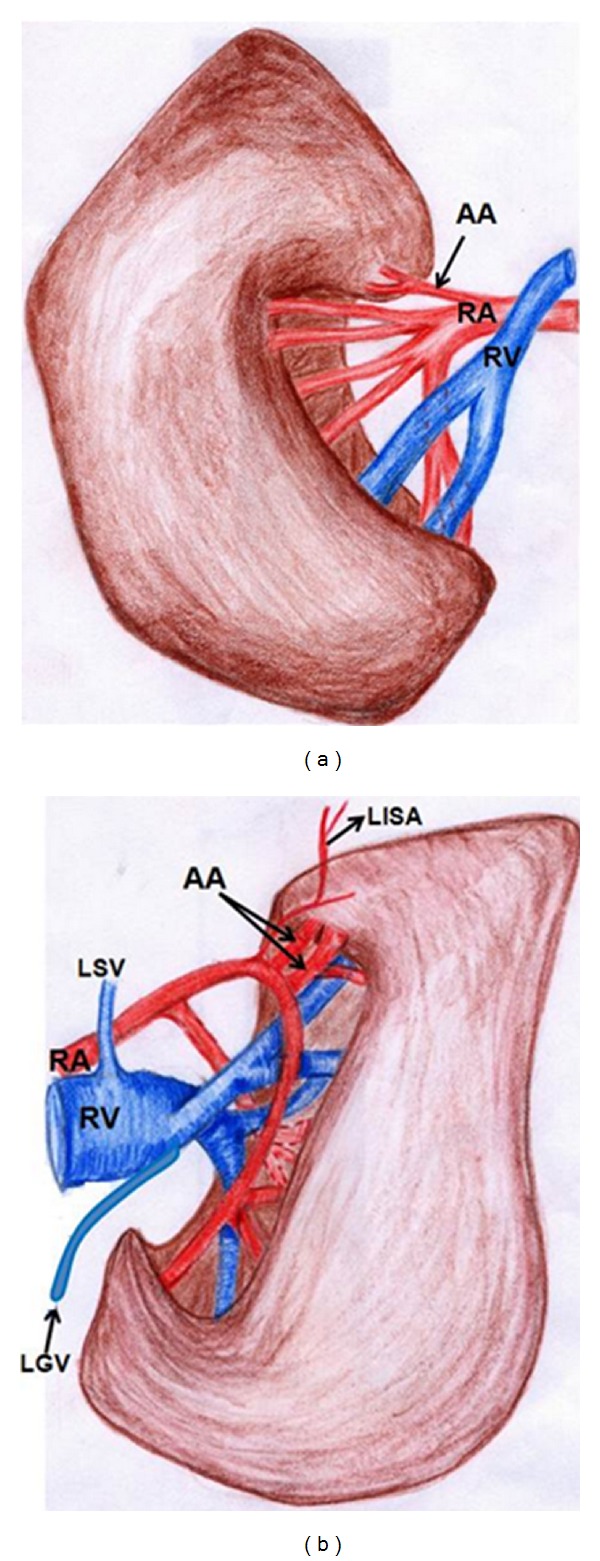
Schematic representation of bilateral renal hilar pattern with distorted shapes of kidneys. AA: aberrant artery, RA: renal artery, RV: renal vein, LGV: left gonadal vein, LSV: left suprarenal vein, and LISA: left inferior suprarenal artery.

The schematic representation of bilateral renal hilar pattern with distorted shapes of kidneys is shown in Figure 4.

## 3. Discussion 

Although abnormal shapes, positions, and vascular variations of the kidney have been reported earlier, to our knowledge, there are no reports on bilateral anomalous variations of the renal vessels as presented in this paper. The variations reported here are peculiar and unique. Variations in the branching pattern of the renal vessels probably might be the cause for the change in the shape of the kidney from the normal bean shape to the retort flask shape which is seen here. Morishima et al. reported a diamond shaped left kidney situated lower than usual, the hilum of which was widely opened and was facing anteriorly [3]. A discoid shaped ectopic kidney located in front of the right common iliac artery has also been reported [4]. This kidney also had associated vascular variations of the renal vessels. 

The abnormalities in the renal arteries are mainly due to the various developmental positions of the kidney [5]. Insufficient degeneration of mesonephric arteries leads to presence of more than one renal artery. Renal artery variations are categorized in to two types: “early branching" and “extra-renal arteries." In early branching main renal artery is more proximal to hilum. Extra-renal arteries are grouped into hilar (accessory) and polar (aberrant) arteries. Hilar arteries enter kidney through hilum with main renal artery; polar arteries penetrate kidney directly through the capsule from outside of the hilum [6, 7]. The aberrant arteries represent the fetal arteries. The persistence of one of the fetal arteries is common (30% of individuals), which usually arises from the aorta to the lower pole of the kidney [8]. But in the present case, neither it is coming from aorta nor is it piercing the lower pole. In the present case, the renal arteries on both the sides divided into subdivisions, some of which again divided into smaller branches before penetrating the hilum and the division of the artery on the left side had an unusual arched course. Also the anterior and posterior divisions of renal vein after emerging separately from hilum of the kidney united to form single trunk on both sides. 

Study conducted by Kaneko et al. presented 25% of multiple renal arteries which included the polar renal arteries [9]. In the literature, significant prevalence of anatomical variations on the left renal vein (about 92%) was found by Baptista-Silva et al., [10], and the presence of multiple right renal veins (more than 2 vessels) was found in about 8% to 9.7% of cases. On the other hand, Bergman et al. pointed that, the renal veins show less variation than do the renal arteries and multiple renal veins to be rare on the left side (1%) and common on the right side (28%) [11]. Senecail et al. described that the anomalies of the renal vein may represent real traps in the interpretation of abdominal imaging, particularly in CT scanning or MRI, where they are not always recognized [12]. The abnormal imaging may be the source of technical difficulties in diagnostic or therapeutic angiography [13]. According to Bayramoglu et al., the variations in the number of renal arterial divisions in the hilar region are generally associated with renal malformations in the embryo [14].

Rouvière et al. observed 29%–65% incidences with anomalous course of renal vessels crossing the renal pelvis cause of ureteropelvic obstruction [15]. Obstruction, strictures, and stenosis may be due to any external compression. Most reliable reason for the extrinsic obstruction by a renal vessel could be attributed to incomplete rotation of the kidney [16]. Hence the rotational defect of the kidney witnesses the anomalous placement of structures in the hilum [17]. Surgical intervention which requires hilar dissection needs separate clamping of the vessels and renal pelvis which is preferred over en bloc mass stapling of renal hilum. A difficult hilar dissection may result in conversion of laparoscopic operation to an open procedure [18]. Anatomical knowledge of distribution of structures in the renal hilum is important for various urological surgical procedures. Thus, the variations described in the current observation present a unique pattern of congenital renal vascular variants having surgical and radiological importance.
